# Combination of metformin and 5-aminosalicylic acid cooperates to decrease proliferation and induce apoptosis in colorectal cancer cell lines

**DOI:** 10.1186/s12885-016-2157-9

**Published:** 2016-02-19

**Authors:** Mona M. Saber, May A. Galal, Afaf A. Ain-Shoka, Samia A. Shouman

**Affiliations:** Pharmacology and Toxicolgy Department, Faculty of Pharmacy, Cairo University, Cairo, 11562 Egypt; Parmacology Unit,Cancer Biology Department, National Cancer Institute, Cairo University, Cairo, 11796 Egypt

**Keywords:** 5-ASA, Inflammation, CRC, NF-κB, STAT3, Metformin

## Abstract

**Background:**

The link between inflammation and cancer has been confirmed by the use of anti-inflammatory therapies in cancer prevention and treatment. 5-aminosalicylic acid (5-ASA) was shown to decrease the growth and survival of colorectal cancer (CRC) cells. Studies also revealed that metformin induced apoptosis in several cancer cell lines.

**Methods:**

We investigated the combinatory effect of 5-ASA and metformin on HCT-116 and Caco-2 CRC cell lines. Apoptotic markers were determined using western blotting. Expression of pro-inflammatory cytokines was determined by RT-PCR. Inflammatory transcription factors and metastatic markers were measured by ELISA.

**Results:**

Metformin enhanced CRC cell death induced by 5-ASA through significant increase in oxidative stress and activation of apoptotic machinery. Moreover, metformin enhanced the anti-inflammatory effect of 5-ASA by decreasing the gene expression of IL-1β, IL-6, COX-2 and TNF-α and its receptors; TNF-R1 and TNF-R2. Significant inhibition of activation of NF-κB and STAT3 transcription factors, and their downstream targets was also observed. Metformin also enhanced the inhibitory effect of 5-ASA on MMP-2 and MMP-9 enzyme activity, indicating a decrease in metastasis.

**Conclusion:**

The current data demonstrate that metformin potentiates the antitumor effect of 5-ASA on CRC cells suggesting their potential use as an adjuvant treatment in CRC.

## Background

Colorectal cancer (CRC) is the third most common cancer with a lifetime risk of 5 %. A functional link between chronic inflammation and cancer has long been suspected but the complete underlying molecular pathways remain unknown [1]. Inflammatory bowel diseases (IBD), including ulcerative colitis (UC) and Crohn’s disease (CD) are chronic inflammatory disorders of the gastrointestinal tract that lead to impairment of the gastrointestinal structure and function [2–4]. Patients suffering from IBD are at an increased risk of developing CRC, this depends on disease duration, as well as, the extent and severity of inflammation [4]. IBD-associated CRC accounts for 1–2 % of all CRC cases, however, IBD patients are six times more likely to die from CRC than the general population [4, 5]. Although carcinogenesis in IBD follows a different sequence of genetic alterations than that observed in sporadic CRC, patients with sporadic CRC have elevated inflammatory cytokine levels indicative of subclinical inflammatory disease [6–8].

Mesalamine [5-aminosalicylic acid (5-ASA)] is the drug of choice in IBD, mainly UC, for maintenance of remission and treatment of mild relapses. It is a weak COX and LOX inhibitor as it is structurally related to NSAIDs, but unlike these compounds it has low systemic resorption and very few side effects even at high doses for long time periods [9]. Epidemiological investigations suggested that long term 5-ASA consumption decreases the risk of developing CRC in IBD patients [10, 11]. In addition, several experimental studies showed that 5-ASA decreases growth and survival of CRC cells [12–15]. The anti-proliferative and pro-apoptotic effects of 5-ASA on several tumor-derived cell lines have been previously reported and different mechanisms have been proposed namely, inhibition of; Wnt/β-catenin pathway [16], EGFR activation [17–19], NF-κB [20], and COX-2 expression [21]. Moreover, Rousseaux et al. showed that mesalamine activates PPAR-y and enhances its expression in CRC cells [22].

Type 2 DM prevalence is estimated to be 8–18 % in newly diagnosed cancer patients, [23]. Evidence indicates that type 2 DM is positively associated with incidence and mortality of CRC with a 30 % increased relative risk compared with non-diabetic individuals [24]. The associated hyperglycemia and hyperinsulinemia result in direct stimulation of cell growth and DNA synthesis along with an increase in pro-inflammatory cytokines production [25]. Metformin, a biguanide derivative, is an oral anti-diabetic drug used as the first line pharmacological treatment in type 2 DM. It acts mainly by inhibiting hepatic glucose production and decreasing peripheral tissue resistance to insulin. This reduces the circulating glucose and insulin levels thus reducing the incidence of diabetes-related complications [26].

Metformin is a safe drug with the most frequent adverse effects being gastrointestinal symptoms but they are usually mild and transient [27]. Many studies revealed the beneficial effect of metformin in decreasing CRC risk [28, 29]. Moreover, it was found to synergistically increase apoptosis of CRC cells in-vitro when combined with other chemotherapy drugs [30, 31].

Since inflammation and hyperglycemia are associated with increased risks of cancer as well as of the major causes of cancer progression, the aim of the present study was to evaluate the effect of combining 5-ASA and metformin on CRC cell lines.

## Methods

### Drugs

Metformin was obtained from CID Co. (Cairo, Egypt). It was freshly dissolved in culture medium, Roswell Park Memorial Institute 1640 (RPMI-1640), as 80 mM stock solution. 5-ASA was kindly provided by Minapharm (Cairo, Egypt) and dissolved in phosphate buffered saline (PBS) just before use. It was added to the medium with the final maximum concentration of PBS 0.1 % v/v, and experiments carried out protected from light.

### Chemicals and antibodies

PBS, RPMI-1640 medium, and sulphorodamine-B (SRB) were all purchased from Sigma Aldrich (St Louis, Missouri, USA). Polyclonal anti-human Bax and Bcl-2 antibodies were obtained from Invitrogen (Carlsbad, CA, USA). Monoclonal anti-human β-actin was obtained from Sigma-Aldrich. All other chemicals were of reagent grade and used without further purification.

### Cell culture

The two available human colorectal cancer cell lines, Caco-2 and HCT-116, were obtained from the American Type Culture Collection (Manassas, USA). They were maintained and grown at the Egyptian National Cancer Institute (Cairo, Egypt) in RPMI-1640 supplemented with 10 % fetal bovine serum, 2 mM L-glutamine, 1.5 g/l sodium bicarbonate and 1 % penicillin/streptomycin. Cells were cultured in a humidified incubator at 37 °C in 5 % carbon dioxide (CO_2_). No ethical approval was required for any aspect of this study.

### Cytotoxicity assay

Cytotoxicity was evaluated using the SRB assay. Briefly, exponentially growing cells were seeded in 96-well microtitre plates at an initial density of 5 × 10^3^ /well. After 24 h, metformin and 5-ASA were added with various concentrations and incubated at 37 °C for 48 h to determine their IC_50_s (the concentration of the drug required to produce 50 % inhibition of cell growth). Cells were fixed with 10 % trichloroacetic acid for 1 h at 4 °C and stained with 0.4 % SRB for 30 min., wells were then washed four times with 1 % acetic acid and air-dried. The dye was solubilized with 10 mM Tris base (pH 10.5) and the optical density (O.D.) was measured spectrophotometrically at 570 nm with the microplate reader (Tecan SunriseTM, Männedorf, Switzerland). The percentage of cell survival was calculated as follows: survival fraction = O.D. (treated cells)/O.D. (control cells).

The IC_50_ values of the two cancer cell lines after 48 h treatment were calculated using sigmoidal dose response curve-fitting models (Graphpad Prism Software, version 5.03, Inc. Avendia de la Playa La Jolla, USA).

Caco-2 and HCT-116 cells were then treated with a combination of subIC_50_ concentrations of metformin and different concentrations of 5-ASA to determine the concentration at which 5-ASA would give a significant difference than metformin alone.

### Oxidative stress markers

Caco-2 and HCT-116 cells were grown in 75 cm^2^ flasks and allowed to adhere for 24 h. Cells were treated with metformin, 5-ASA or the combination of both substances for 48 h then collected by trypsinisation. The cell pellet was washed twice with PBS. To the cell suspension 1 ml of 20 % (w/v) trichloroacetic acid (TCA; Sigma, USA) containing 0.8 % (w/v) thiobarbituric acid (TBA; Sigma, USA) was added and mixed well. MDA (decomposition product of the lipid peroxidation process) level was determined colorimetrically by measuring the pink pigment product resulting from the reaction of one molecule of MDA with two molecules of TBA at 535 nm. Protein was measured by the method of Bradford and MDA is expressed in nmol MDA per mg protein. For determination of total SH group, protein precipitation was carried out using 10 % TCA then samples were centrifuged at 3000 rpm for 10 min at 4 °C. The resultant supernatant was mixed with phosphate buffer and Ellman’s reagent (Sigma-Aldrich, Milan, Italy). The method depends on the reduction of thiol reagent; Ellman’s reagent by the sulfhydryl SH group in GSH to form the yellow chromophore; 5-thionitrobenzoic acid, measured spectrophotometrically at 412 nm. GSH is expressed in nmol GSH per mg protein.

### Real-time PCR analysis

Analysis of COX-2, IL-1β, IL-6, TNF-α, TNF-R1, and TNF-R2 RNA expression was performed by real-time PCR. Caco-2 and HCT-116 cells, treated with metformin and 5-ASA, were collected by trypsynisation. Total RNA was extracted from cells using TRIzol reagent (Invitrogen, Milan, Italy), according to the manufacturer’s instructions. Concentration and purity of the RNA was checked by A_260_/A_280_ optical density ratio. RNA (1 μg/sample) was retro-transcribed into complementary DNA (cDNA) and 1 μl of cDNA/sample was then amplified using the following conditions: denaturation 1 min at 95 °C, annealing 30s at 60 °C for COX-2, TNF-R2, IL-1β, and β-actin or 30s at 57 °C for TNF-α, TNF-R1 and IL-6, followed by 30s of extension at 72 °C. Primers sequence was as shown in Table 1 and they were obtained from Invitrogen (Milan, Italy). RT-PCR was performed using the IQ SYBER Green Supermix (Bio-Rad Laboratories, Milan, Italy). mRNA levels were calculated relative to β-actin, which was unaffected by metformin and 5-ASA treatment.

**Table 1:** Primer sequences

Gene	Primer
COX-2	FWD: 5′-CCC TTC CTT CGA AAT GCA AT-3′ REV: 5′-CAT TTG AAT CAG GAA GCT GC-3′
IL-1β	FWD: 5′-GGA CAA GCT GAG GAA GAT GC-3′ REV: 5′-TTT TTT GCT GTG AGT CCC GG-3′
IL-6	FWD: 5′-GAG ACT TGC CTG GTG AAA AT-3′ REV: 5′-CAG GGG TGG TTA TTG CAT CT-3′
TNF-α	FWD: 5′ ACA AGC CTG TAG CCC ATG TT-3′ REV: 5′ AAA GTA GAC CTG CCC AGA CT-3′
TNF-R1	FWD: 5′-CGC TTC AGA AAA CCA CCT CAG AC-3′ REV: 5′-CCA AAG AAA ATG ACC AGG GGC-3′
TNF-R2	FWD: 5′-GCT CTG ACC AGG TGG AAA CTC AAG-3′ REV: 5′-GGA TGA AGT CGT GTT GGA GAA CG-3′
β-actin	FWD: 5′-TCT GGC ACC ACA CCT TCT ACA ATG-3′ REV: 5′-AGC ACA GCC TGG ATA GCA ACG-3′

### Western blot analysis

Aliquots of protein supernatants containing equal amounts of protein and sodium dodecyl sulphate (SDS) reducing buffer were boiled for 5 min. They were then electrophoresed on SDS-polyacrylamide gels and transferred to polyvinyldiene difluoride membranes. The membranes were blocked with 5 % non-fat dry milk and probed with specific primary antibodies of monoclonal anti-Bax and Bcl2 antibodies followed by incubation with peroxidase-conjugated secondary antibodies. The blots were developed with Amersham ECL western blotting kit (GE Healthcare, Amersham Place, Little Chalfont, U.K) according to the manufacturer’s instructions. The blots were quantified by ChemiDoc XRS 4.6.9 (Bio-Rad Laboratories Inc., Hercules, CA, USA.) software and protein loading was corrected for β-actin as loading control.

### ELISA techniques

The different proteins were determined in both cell lines according to the kit manufacturer’s instructions. For NF-κB the Kamiya Biomedical assay kit (Seattle, USA) was used, while the RayBiotech (Georgia, USA) was used for TNF-α, IL-6, STAT3, MMP-2 and −9. The assay employs the quantitative sandwich enzyme immunoassay technique. A monoclonal antibody specific for human NF-κB, TNF-α, IL-6, STAT3, MMP-2 or −9 has been pre-coated onto a microplate. Samples were pipetted into the wells and the measured human biomarkers present in the solutions were bound by the immobilized antibody. A yellow color is developed which is proportional to the amount of NF-κB/ TNF-α/IL-6/ STAT3/MMP-2/MMP-9 bound. The intensity of the color is measured at 450 nm.

Caspase-3 activity was measured based on spectrophotometric detection of the chromophore p-nitroaniline (pNA) at 405 nm after cleavage from its labelled substrate DVD-pNA. Protein concentration of the samples was analyzed and normalized in lysis buffer to equal protein concentrations. Colorimetric assay (Caspase-3/CPP32, BioVision, Milpitas, USA) was used according to the manufacturer’s instructions.

### Scratch wound healing assay

Caco-2 and HCT-116 cells were grown in 6 well plates and allowed to adhere for 24 h. Gently and slowly the monolayer was scratched in one direction with a new 1 ml pipette tip across the center of the well. The resulted gap distance therefore equals to the outer diameter of the end of the tip. The wells were then washed twice with medium to remove the detached cells. Cells were treated with metformin, 5-ASA or the combination of both substances for 48 h. Cells were washed twice with 1x PBS, then fix the cells with 3.7 % paraformaldehye for 30 min and they were stained with 1 % crystal violet in 2 % ethanol for 30 min. Photos were taken for the stained monolayer on a microscope. The gap distance was measured using the Leica Qwin-Plus software (Leica Microsystems, UK).

### Statistical analysis

All the data are expressed as mean ± SD from three different experiments and comparisons between means were carried out using one way analysis of variance (ANOVA) followed by Tukey-Kramer multiple comparisons test. A probability level of less than 0.05 was accepted as being significant in all types of statistical tests. All statistical analysis was performed using GraphPad InStat, version 5.0 (GraphPad, San Diego, California, USA).

## Results

### Co-incubation of metformin enhanced 5-ASA-mediated inhibition of cell counts in Caco-2 and HCT-116 cells

**Fig. 1:**
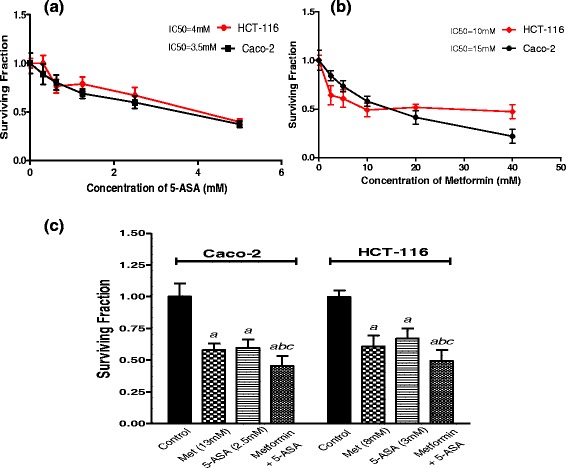
Effect of different concentrations of (**a**) 5-ASA and (**b**) Metformin on surviving fractions of Caco-2 and HCT-116 cells treated for 48 h. **c** Surviving fraction of Caco-2 and HCT-116 cells after treatment with subIC_50_ concentrations of metformin, 5-ASA and a combination of both for 48 h. All data are expressed as mean ± SD of 3 separate experiments performed in triplicates. The statistical significance of the results was analyzed using one way ANOVA followed by Tukey-Kramer multiple comparison test. ^***a***^ Significantly different from control, ^***b***^ from metformin and ^***c***^ from 5-ASA (*P* <0.05)

Caco-2 cells were treated with 13 mM metformin, 2.5 mM 5-ASA, or the combination of both for 48 h. Similarly, HCT-116 cells were treated with 8 mM metformin, 3 mM 5-ASA, or the combination of both for 48 h. Significant inhibition of cell proliferation were seen in all treatment groups with the combination group showing the highest inhibition reaching 55 % of the control compared to nearly 40 % reached by the solo treatments in both cell lines Fig. 1.

### Exaggerated increase in oxidative stress upon combined treatment with metformin and 5-ASA

**Fig. 2:**
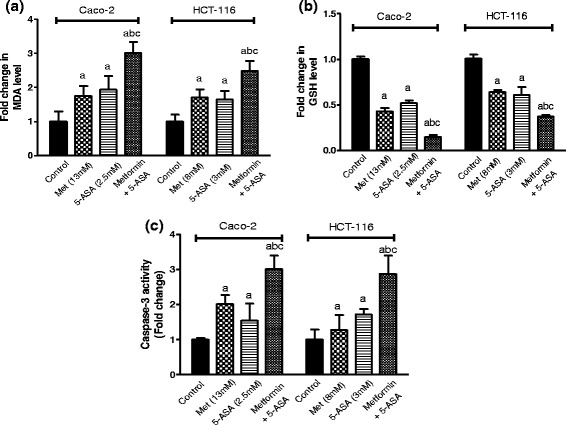
Effect of treatment of Caco-2 and HCT-116 cells for 48 h with subIC_50_ concentrations of metformin, 5-ASA or the combination of both drugs on oxidative stress markers measured as (**a**) MDA level (**b**) GSH level and on (**c**) caspase-3 activity. Data are indicative of 3 separate experiments. The statistical significance of the results was analyzed using one way ANOVA followed by Tukey-Kramer multiple comparison test. ^***a***^ Significantly different from control, ^***b***^ from metformin and ^***c***^ from 5-ASA (*P* <0.05)

Combination of subIC_50_ concentrations of both drugs produced a pronounced increase in MDA level (Fig. 2a) and a greater decrease in the intracellular GSH level than each drug alone (Fig. 2b). In Caco-2 cells the combination of metformin and 5-ASA resulted in a significant increase in MDA level of 3 folds compared to, 1.9 and 1.75 folds produced by either of the treatments. In addition, a reduction of GSH exceeding 85 % was observed compared to nearly 50 % decrease produced by each drug alone. A similar effect was produced when both drugs were added to the HCT-116 cells where MDA level showed an elevation mounted to 2.5 folds compared to nearly 1.7 fold increase produced by solo treatments. Moreover, intracellular GSH depletion reached 65 %, while only a 35 % decrease in GSH levels was observed after adding metformin or 5-ASA alone.

### Co-incubation of metformin enhances 5-ASA-induced apoptosis

The addition of metformin to 5-ASA succeeded in activating the caspase-3 enzyme more than the individual treatments in both cell lines reaching 3 fold the control group (Fig. 2c). Solo treatments in Caco-2 and HCT-116 cells increased active caspase-3 to levels ranging from 1.2–1.7 folds.

**Fig. 3:**
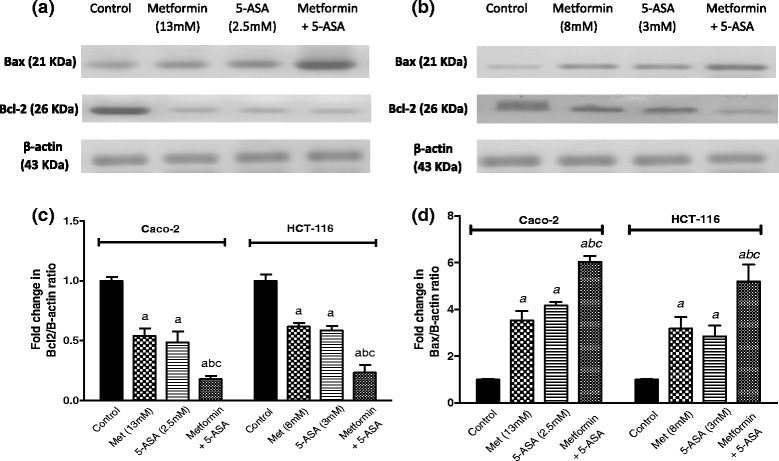
Western blot for Bcl-2 and Bax levels in (**a**) Caco-2 and (**b**) HCT-116 cells after treatment with metformin, 5-ASA or their combination. One representative western blot of three independent experiments is shown. Effect of treatment of Caco-2 and HCT-116 cells for 48 h with subIC_50_ concentrations of metformin, 5-ASA or the combination of both drugs on (**c**) Bcl-2 level (**d**) Bax level. All data are expressed as mean ± SD of 3 separate experiments. The statistical significance of the results was analyzed using one way ANOVA followed by Tukey-Kramer multiple comparison test. ^***a***^ Significantly different from control, ^***b***^ from metformin and ^***c***^ from 5-ASA (*P* <0.05)

The increase in caspase-3 activity was accompanied by increased apoptotic Bax levels and decreased expression of the anti-apoptotic Bcl-2 protein in both cell lines (Fig. 3), following treatment with 5-ASA, metformin or their combination after 48 h. The combination group showed the most significant change in Bax levels, as compared to individual treatments where it produced a 16 and 13 folds increase in Caco-2 and HCT-116 cells, respectively. On the other hand, the anti-apoptotic Bcl-2 expression decreased by 85 % and 80 % in Caco-2 and HCT-116 cells after combination treatment.

### Combination of metformin and 5-ASA downregulates TNF-α, TNF-α receptors (TNF-R1 and TNF-R2), and IL-1β and inhibits the activation of NF-κB

**Fig. 4:**
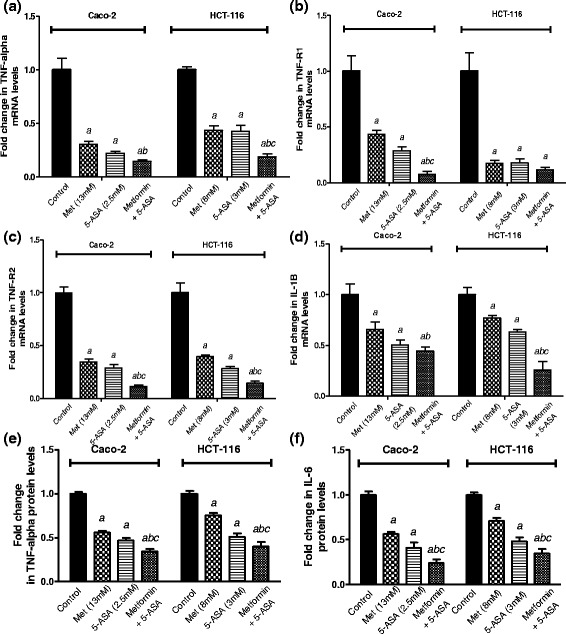
Effect of treatment of Caco-2 and HCT-116 cells for 48 h with subIC_50_ concentrations of metformin, 5-ASA or the combination of both drugs on (**a**) TNF-α (**b**) TNF-R1 (**c**) TNF-R2 and (**d**) IL-1β gene expression levels and on (**e**) TNF-α and (**f**) IL-6 protein levels. All data are expressed as mean ± SD of 3 separate experiments. The statistical significance of the results was analyzed using one way ANOVA followed by Tukey-Kramer multiple comparison test. ^***a***^ Significantly different from control, ^***b***^ from metformin and ^***c***^ from 5-ASA (*P* <0.05)

All treatment groups produced a prominent decrease in the expression of TNF-α and its receptors (Fig. 4). In Caco-2 cells, the combination group did not significantly differ from the 5-ASA group although it produced 85 % decrease in TNF-α expression while 5-ASA produced 80 % decrease. However, in HCT-116 cells the combination of both drugs downregulated TNF-α expression by 80 % compared to 60 % decrease produced by either drug. These results were in agreement with the protein levels of TNF-α that decreased significantly after the combination treatment compared to either of the drugs alone in both cell lines as shown in Fig. (4e). Moreover, following treatment of Caco-2 cells with the two drugs, there was a downregulation of both TNF-α receptors that was greater and statistically significant compared to the solo drug treatments where TNF-R1 and TNF-R2 gene expression levels decreased by 95 % and 90 % compared to the control group. In addition, the combination group in HCT-116 cells decreased TNF-R2 expression level reaching solely 10 % compared to the untreated cells.

Concerning IL-1β gene expression level, after treatment of Caco-2 and HCT-116 cells with a combination of metformin and 5-ASA, IL-1β levels decreased by 55 % compared to the control (Fig. 4d). This decrease was statistically significant from the metformin group but not the 5-ASA group. On the other hand, a 75 % decrease was observed in the HCT-116 cells that was significant from all treatment groups.

**Fig. 5:**
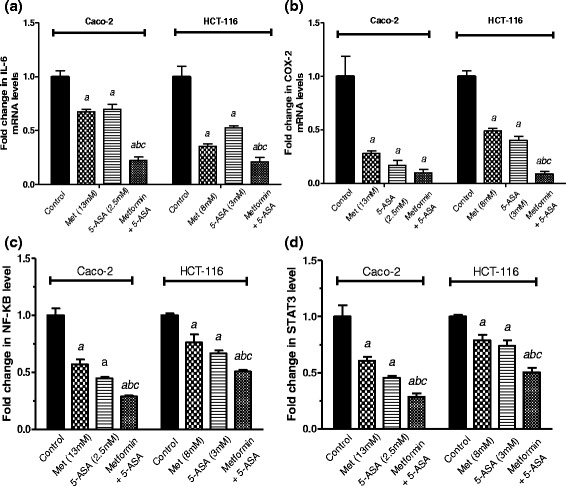
Effect of treatment of Caco-2 and HCT-116 cells for 48 h with subIC_50_ concentrations of metformin, 5-ASA or the combination of both drugs on (**a**) IL-6 and (**b**) COX-2 gene expression levels. Fold change in levels of (**c**) NF-κB and (**d**) STAT3 transcription factors in Caco-2 and HCT-116 cells after 48 h of treatment with metformin, 5-ASA or their combination. All data are expressed as mean ± SD of 3 separate experiments. The statistical significance of the results was analyzed using one way ANOVA followed by Tukey-Kramer multiple comparison test. ^***a***^ Significantly different from control, ^***b***^ from metformin and ^***c***^ from 5-ASA (*P* <0.05)

In parallel with the previous results, NF-κB levels (Fig. 5c) showed significant decrease to 29 % and 51 % in Caco-2 and HCT-116 cells upon the addition of metformin to 5-ASA.

### Intensified inhibition of IL-6 gene expression and inhibition of STAT3 activation upon addition of metformin to 5-ASA

The expression of IL-6 (Fig. 5a) after exposure to metformin or 5-ASA was decreased significantly by 30 % for both drugs in the Caco-2 cell line. On HCT-116 cells, metformin and 5-ASA produced a 65 % and 50 % decrease in IL-6 expression, compared to the control. Furthermore, the combination resulted in an exaggerated decrease of 80 % in IL-6 expression compared to either drug alone in both cell lines. Confirming the gene expression results, the IL-6 protein level showed a significant decrease after treatment in both cell lines than the solo treatments as shown in Fig. (4f).

As illustrated in Fig. (5d) the greatest inhibition of STAT3 activity was observed in the combination group reaching 29 % and 50 % in Caco-2 and HCT-116 cell lines.

### Metformin exaggerates the 5-ASA-induced inhibition of COX-2 enzyme gene expression

The expression of COX-2 gene, after treatment of Caco-2 cells with metformin decreased by 75 % compared to control, while treatment with the anti-inflammatory agent 5-ASA caused a further downregulation in the gene expression by 85 % (Fig. 5b). The COX-2 gene expression reached its maximum inhibition (90 %) when the Caco-2 cells were treated by both agents.

The same pattern was reflected on the HCT-116 cells, where the COX-2 gene expression was downregulated by metformin, 5-ASA and their combination in this descending order by 50 %, 60 % and 90 %, compared with the control group.

### Pronounced suppression of matrix metalloproteinase-2 and −9 and inhibition of cell migration after combining metformin and 5-ASA

**Fig. 6:**
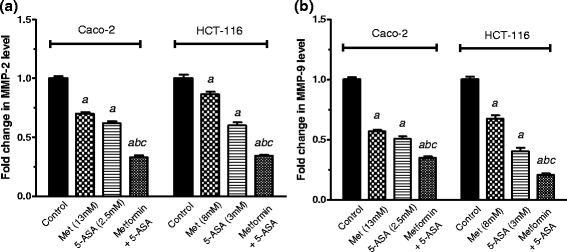
Fold change in (**a**) MMP-2 and (**b**) MMP-9 enzyme levels after 48 h of treatment of Caco-2 and HCT-116 cells with metformin, 5-ASA or their combination. All data are expressed as mean ± SD of 3 separate experiments. The statistical significance of the results was analyzed using one way ANOVA followed by Tukey-Kramer multiple comparison test. ^***a***^ Significantly different from control, ^***b***^ from metformin ^***c***^ from 5-ASA (*P* <0.05)

**Fig. 7:**
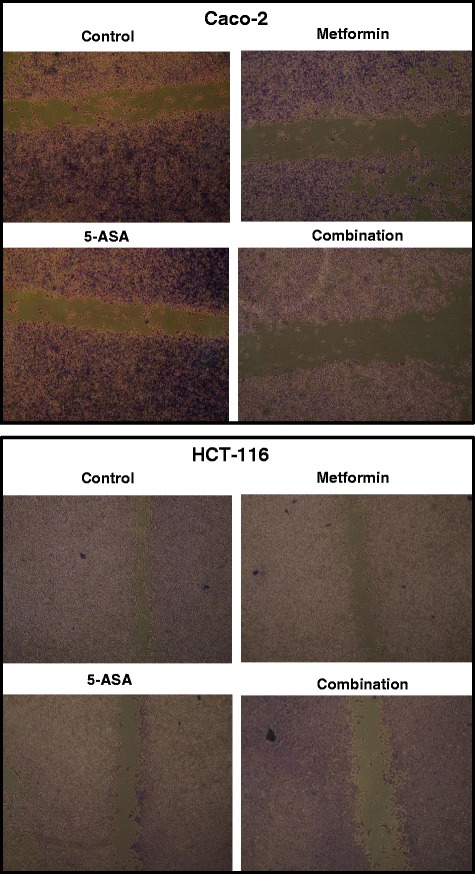
Photos of the stained monolayer of Caco-2 and HCT-116 cells without treatment and after treatment for 48 h with subIC_50_ concentrations of metformin, 5-ASA or the combination of both drugs

The addition of metformin to 5-ASA in Caco-2 cells significantly lowered MMP-2 and −9 levels to 33 % and 35 % respectively (Fig. 6). Similarly, treatment of the HCT-116 cells with a combination of both drugs decreased MMP-2 and −9 enzyme levels to 34 % and 21 % compared to the control. The results of the scratch wound healing assay were in accordance with the MMP levels as the combination of both drugs resulted in a 25 % decrease from control at zero time in Caco-2 cells and a 20 % inhibition in HCT-116 cells. Moreover the results of this assay confirm the fact that HCT-116 cells are highly tumorigenic and metastatic Fig. 7.

## Discussion

In the current study, we used 2 colorectal cancer cell lines Caco-2 and HCT116. Caco-2 cell line is colorectal adenocarcinoma cells with intermediate characteristics, as it is spontaneously differentiating tumor in normal culture, it is tumorigenic but hasn’t a metastatic behavior in vivo.. Treatment with very low drug concentrations of metformin, 5-ASA and their combination was coupled by a significant decrease in the surviving fraction of CRC cells. The increased cell death could be explained by intracellular GSH depletion and increase in MDA level, indicating that cell death could be initiated or underwent under oxidative stress originating from modulation of the intracellular redox system. Our results are in accordance with previous studies showing that 5-ASA or metformin can increase oxidative stress in certain types of cancer thus leading to increased apoptosis [12, 32].

Another explanation for the increased apoptosis is the inhibition of inflammation. The central players involved in inflammation-mediated tumor progression include IL-1β, IL-6 and TNF-α [33]. Data from several inflammation-associated cancer models implicate these inflammatory cytokines in being the bridge between inflammation and tumorigenesis [34]. On CRC cells, addition of metformin to 5-ASA significantly reduced the gene expression of TNF-α and its receptors, TNF-R1 and TNF-R2.

Altered expression of the genes coding for TNF-α and its receptors was observed in neoplastic diseases [35]. In CRC, malignant cell derived TNF-α, enhances the growth and metastasis of the tumor as evidenced from animal models [36]. Chronic TNF-α production by malignant or host cells or both may directly contribute to oncogene activation, DNA damage and metastasis [37]. The main receptor mediating TNF-α effects is TNF-R1 which when stimulated, induces activation of NF-κB after a series of intracellular events [38]. On the other hand, the increased TNF-α level could be due to the continuous activation of NF-κB [39]. NF- κB activation has long been known to suppress apoptosis as it promotes transcription of anti-apoptotic genes, one of which is Bcl-2 [40]. The decline in TNF-α or TNF-R1 genetic expression will therefore decrease NF-κB activation and thus increase apoptosis. In our study, combination of 5-ASA and metformin resulted in the greatest inhibition in the TNF-α and TNF-R1 gene expression, and NF- κB protein level with a subsequent increase in apoptosis, evident by decreased level of Bcl-2 protein expression.

Although TNF-α mediates its action mainly through TNF-R1 activation, TNF-R2 has been linked to increased colon cancer cell growth and proliferation [41, 42]. It was found that both IL-6 and TNF-α interact to induce TNF-R2 expression and function in colon cancer cells, suggesting that a specific microenvironment of multiple cytokines is required to induce TNF-R2, as found in IBD or IBD associated CRC.

Our study revealed a pronounced down-regulation of TNF-R2 gene expression in Caco-2 and HCT-116 cell lines in all treatment groups with the greatest effect being that of the combination group. There were no previous studies conducted on 5-ASA or metformin that assessed their effect on TNF-R2 expression. In this study evidence of the effect of both drugs either alone or in combination on TNF-R2 expression levels was demonstrated.

Together with TNF-α, IL-1β also increases NF-κB transcription thus increasing tumor adhesiveness, invasion and angiogenesis [43]. Likewise, activation of NF-κB results in an increase in IL-1β [39]. Polymorphisms of IL-1β are associated with increased cancer risk [44]. Caco 2 cells expresses active COX 2 and epidermal growth factor receptor (EGFR). On the other hand HCT-116 cell line are undifferentiated colorectal carcinoma cells that don’t express COX2 and are positive for transforming growth factor beta 1 (TGF beta 1) and beta 2 (TGF beta 2) expression. In the current study, combining metformin with 5-ASA showed a remarkable decrease in IL-1β gene expression in both cell lines respectively. The decrease in NF-κB protein level, cell proliferation, increase in apoptosis and decrease in MMP-2 and −9 expression, all support the inhibition of the downstream signaling pathway of IL-1β.

Adding to the panoply of molecules, IL-6 is one of the best characterized pro-tumorigenic cytokines. IL-6 is not only produced by immune cells but also by epithelial and malignant cells. Its production is induced by a variety of stimuli one of which is NF-κB. Therefore, IL-6 production can be stimulated indirectly by TNF-α or IL-1β that activate the NF-κB or directly by PGE2 [45]. IL-6 promotes colon cancer cell proliferation, survival, migration, invasion, metastasis, angiogenesis and inflammation [46]. These effects are a result of the activation of the downstream target of IL-6, the STAT3 transcription factor. Persistent activation of STAT3 has been reported in a variety of human tumors, including the colon [47]. This persistent activation can also be the reason of increased interleukins levels [48].

The current study revealed that addition of metformin to 5-ASA resulted in a prominent decrease in IL-6 mRNA levels that was accompanied by a decrease in STAT3 level and therefore increased cell death. It is demonstrated herein that the combination group expressed the lowest Bcl-2 and highest Bax protein levels. This was associated with increased caspase-3 activity and apoptosis in both cell lines. This effect may be attributed in part to the suppression of IL-6 and STAT3.

COX-2 is another important pro-inflammatory mediator that is implicated in the process of carcinogenesis. It was shown to be upregulated early in CRC and plays a major role in its progression [49] by regulating the process of proliferation, angiogenesis and metastasis [50]. It was previously demonstrated that one of the mechanisms of 5-ASA to decrease proliferation was by inhibiting COX-2 enzyme [21]. Metformin as well, has been reported to inhibit inflammatory responses and COX-2 expression [51], however, its effect on COX-2 expression in CRC cells has not been clarified. Our data show that combination of both drugs resulted in an exaggerated inhibition of COX-2 expression than that produced by the solo treatments suggesting a synergistic effect. This explains the inhibition of MMP’s level that was observed in the combination group since COX-2 overexpression increases invasiveness of CRC cells by inducing MMP expression [52]. It was reported that COX-2 inhibitors decrease MMP-2 and −9 expression [53]. Therefore, the decrease in MMP-2 and −9 level could be due to inhibition of COX-2 and the NF-κB and STAT3 signaling pathways. Several studies showed that metformin or 5-ASA can inhibit MMP’s expression [54–56].

## Conclusions

**Fig. 8:**
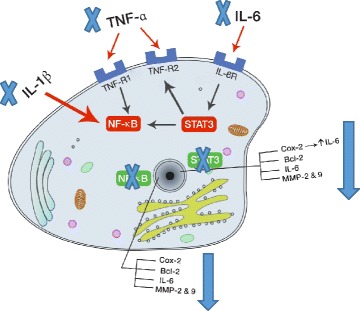
Addition of 5-ASA and metformin on CRC cells inhibit the NF-κB and STAT3 signaling pathways. Inhibition of gene expression of IL-1β and TNF-α decreases activation of NF-κB resulting in inhibition of target gene expression. Moreover, decrease in IL-6 mRNA levels reduces STAT3 activation and thus decreasing target gene transcription. The target genes for both pathways include COX-2, Bcl-2, IL-6 and MMP’s. Low levels of COX-2 and IL-6 gene expression and Bcl-2, MMP-2 and −9 protein levels confirm the inhibition of both the signaling pathways

Our data (Fig. 8) clarify that treatment of CRC cells with a combination of 5-ASA and metformin increases cell death than either drug alone. This increase in apoptosis may be due to inhibition of the STAT3 and NF-κB signaling pathways. The decreased level of cytokines that stimulate those pathways (TNF-α, IL-1β and IL-6), lowered protein levels of the activated transcription factors (STAT3 and NF-κB) and decreased protein or expression levels of their target genes (TNF-α, IL-6, COX-2, MMP-2, MMP-9 and Bcl-2) confirm the inhibition of the key pathways in inflammation-mediated tumor promotion and progression.

## Abbreviations

COX: cyclooxygenase
CRC: colorectal cancer
DM: diabetes mellitus
EGFR: Epidermal growth factor receptor
GSH: glutathione
IBD: inflammatory bowel disease
IL-1β: Interleukin-1β
IL-6: Interleukin-6
LOX: Lypooxygenase
MDA: malondialdehyde
MMP: matrix metalloproteinase
NF-κB: nuclear factor kappa B
NSAIDs: Non-steroidal anti-inflammatory drugs
O.D.: optical density
PPAR-y: peroxisome-proliferator-activated receptor gamma
RPMI-1640: Roswell Park Memorial Institute 1640
STAT3: signal transducer and activator of transcription 3
TNF-R: Tumor necrosis factor receptor
TNF-α: Tumor necrosis factor-α
